# The posterior cruciate ligament angle in the setting of anterior cruciate ligament deficient knees: the effect of gender, age, time from injury and tibial slope

**DOI:** 10.1007/s11547-025-01951-x

**Published:** 2025-01-25

**Authors:** Fabrizio Di Maria, Riccardo D’Ambrosi, Luca Maria Sconfienza, Stefano Fusco, Elisabeth Abermann, Christian Fink

**Affiliations:** 1 Department of General Surgery and Medical Surgical Specialties, Section of Orthopaedics and Traumatology, University Hospital Policlinico “Rodolico-San Marco” University of Catania, Catania, Italy; 2 IRCCS Istituto Ortopedico Galeazzi – Sant’Ambrogio, Milan, Italy; 3 Dipartimento di Scienze Biomediche per la Salute, Università degli Studi di Milano, Milan, Italy; 4 Gelenkpunkt-Sports and Joint Surgery FIFA Medical Centre of Excellence, Innsbruck, Austria; 5 Research Unit for Orthopaedic Sports Medicine and Injury Prevention (OSMI), Private University for Health Sciences Medical Informatics and Technology, Innsbruck, Austria

**Keywords:** Anterior cruciate ligament, MRI, Posterior cruciate ligament, PCL, Tibial slope

## Abstract

**Purpose:**

This study aimed to assess the posterior cruciate ligament (PCL) angle in anterior cruciate ligament (ACL) deficient knees and correlate it with anatomical and demographic factors such as tibial slope, anterior tibial translation, age, gender, and time of injury.

**Material and methods:**

Patients were eligible for inclusion if they were clinically diagnosed with an ACL tear confirmed by MRI. For each patient, the following parameters were evaluated: PCL angle (PCLA), medial tibial slope (MTS), lateral tibial slope (LTS), medial anterior tibial translation (MATT), and lateral anterior tibial translation (LATT).

**Results:**

A total of 193 patients were included in the study, comprising 91 (47.2%) females and 102 (52.8%) males, with a mean age of 30.27 ± 12.54 years. The mean time from injury to MRI was 14.18 ± 55.77 days. In the overall population, the mean PCL angle was 128.72 ± 10.33°, the mean medial tibial slope was 3.57 ± 2.33°, and the mean lateral tibial slope was 6.07 ± 3.52°. The mean medial and lateral anterior tibial translations were 4.76 ± 2.02 mm and 7.01 ± 2.48 mm, respectively. In 190 cases (98.4%), the PCL angle was ≥ 105°. The PCL angle negatively correlated with medial and lateral anterior tibial translation (*p* < 0.05). Females exhibited a higher PCL angle compared to males (*p* = 0.019).

**Conclusion:**

In the context of ACL lesions, the PCL angle has a normal value in acute injuries (> 105°) and decreases over time. The PCL angle is negatively correlated with anterior tibial translation, and females have a higher PCL angle compared to males.

**Level of evidence IV:**

Retrospective Cohort.

**Supplementary Information:**

The online version contains supplementary material available at 10.1007/s11547-025-01951-x.

## Introduction

Diagnosing an anterior cruciate ligament (ACL) injury and developing a treatment plan rely heavily on the results of magnetic resonance imaging (MRI) and the assessment of knee joint laxity during physical examination. MRI is the most effective method for detecting structural integrity and identifying the stigmata of injury [1–3].

However, the extent of laxity should be assessed through physical examination, including the Lachman and pivot shift tests. The correlation between imaging and physical examination findings is crucial, as the diagnosis of ACL rupture and the decision to proceed with surgery depend on both the ligament's disruption and the resulting laxity [4, 5]. Various MRI findings, both primary and secondary, can indicate ACL damage. However, the likelihood of each result being positive may differ depending on the time elapsed since the injury occurred until the MRI is performed [6, 7]. A significant amount of knee joint laxity following an ACL injury is likely due to damage to the ligament itself [6]. Key observations such as the inability to visualize certain structures breaks in continuity, unusual signal strength, and irregular ligament morphology may be associated with the extent of looseness in the knee joint. Furthermore, bone contusions, a Segond fracture, the deep sulcus sign, a diminished posterior cruciate ligament (PCL) angle, and anterior translation of the tibia in relation to the femur are ancillary findings that may suggest knee joint laxity. The initial three observations may signify the injury's severity, while the subsequent two may be directly attributable to the anterior dislocation of the tibia [7–9].

The buckling of the PCL can occur in both healthy persons and those with ACL tears. Numerous studies have demonstrated a correlation between the PCL's arcuate morphology and chronic ACL ruptures, as opposed to acute ruptures. The PCL angle is utilized to assess the degree of ligament buckling, defined as the angle formed between two lines extending through the tibial and femoral insertions of the PCL. An angle of 105° is recognized as indicative of PCL buckling and serves as an indirect predictor of ACL rupture [10, 11].

Limited literature is available on the degree of concomitant PCL angle and its correlations with demographic factors such as age, gender, chronicity of the injury, and other radiologic parameters (tibial slope and anterior tibial translation).

Therefore, this study aimed to assess the PCL angle in ACL-deficient knees and correlate it with anatomical and demographic factors such as tibial slope, anterior tibial translation, age, gender, and time of injury.

## Material and methods

Following Institutional Review Board approval (ACL-L2104), two authors examined the MR scans of all patients diagnosed clinically with ACL injury.

This study was executed in accordance with the Strengthening the Reporting of Observational Studies in Epidemiology (STROBE) declaration [12]. All procedures were executed in compliance with the criteria outlined in the 1964 Helsinki Declaration and its subsequent revisions [13].

Patients were eligible for inclusion if they had a clinical diagnosis of an ACL injury, corroborated by MRI. Patients were excluded from the trial if they had a prior surgical history on the index knee, concurrent injuries including tibial or femoral fractures, complete tears of the posterior cruciate ligament, quadriceps or patellar tendon, intra-articular infections, or malignancies.

Patients with low-quality preoperative MRI data were removed due to motion artifacts, insufficient magnetic field strength (minimum 1.5 T), or inadequate resolution that hindered proper imaging of the posteromedial knee anatomy and anterolateral ligament. Baseline demographic information, such as patient age, sex, and duration from injury to MRI, were documented manually. An interval of 90 days from injury to MRI was deemed indicative of a chronic ACL lesion [14].

### MRI protocol

All MRI scans were performed on a high-field MRI scanner (≥ 1.5 T) using a standard knee protocol including at least four sequences (slice thickness ≤ 3 mm): sagittal, coronal, and axial fat-suppressed proton-density weighted, and sagittal T1-weighted [15].

### Radiologic assessment

Every single image were evaluated separately by two musculoskeletal radiologists. A combined, second-phase MRI evaluation was conducted to achieve consensus in cases of discrepancies after 4–6 weeks. All appropriate MRI scans were imported into a DICOM medical imaging viewer (Horos v3.0, Horos project, Annapolis, MD) to conduct measurements with digital tools with an accuracy of 1.0 mm [16].

For each patient, the following parameters were evaluated:

- PCL angle (PCLA)
- Medial tibial slope (MTS)
- Lateral tibial slope (LTS)
- Medial anterior tibial translation (MATT)
- Lateral anterior tibial translation (LATT)

**Fig. 1:**
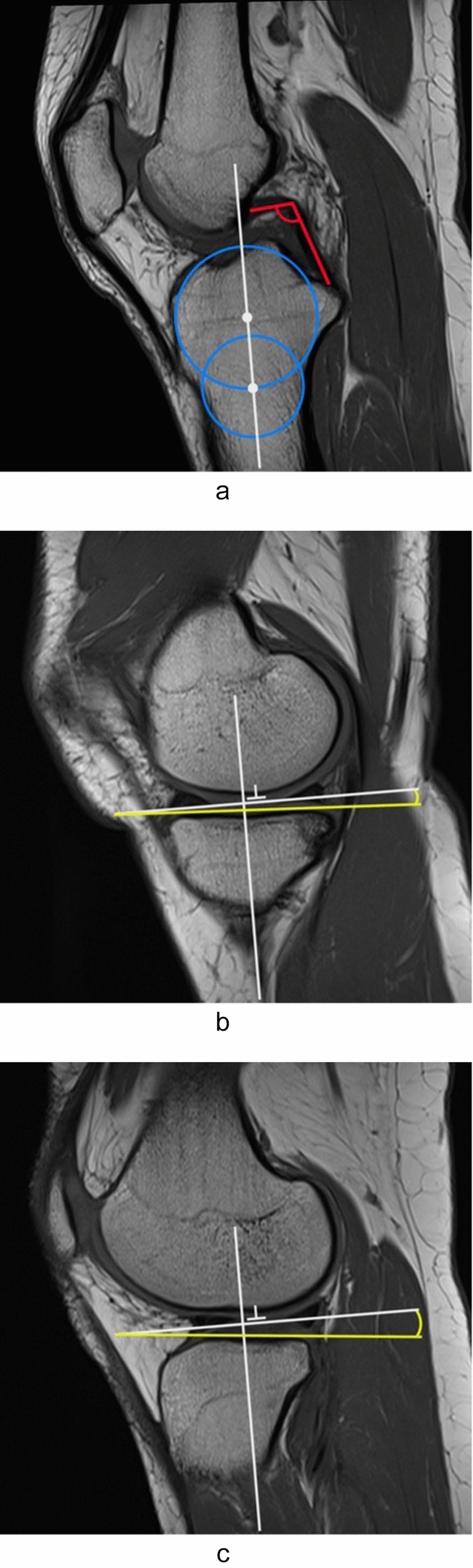
**a** Central sagittal slice of a T1-weighted knee MRI. The posterior cruciate ligament angle (PCLA) is formed by two lines drawn through the central portion of the tibial insertion and the femoral insertion of the posterior cruciate ligament (red lines). Blue circles represent the basis for the assessment of the tibial longitudinal axis (the white line connecting the centers of the two circles). **b, c**) Assessment of medial **b** and lateral **c** tibial slope. The tibial slope corresponds to the angle formed by the orthogonal line to the longitudinal axis (white horizontal line) and the tangent line (yellow line) to the center of the medial tibial **b** and lateral **c** tibial plateau

The angle of the posterior cruciate ligament was delineated, as per McCauley et al., as the angle formed between a line extending through the central region of the tibial insertion of the posterior cruciate ligament and a line extending through the central region of the femoral insertion of the posterior cruciate ligament (Fig. 1a) [17].

The medial and lateral tibial slopes were measured via MRI, as per Hudek et al. [14]. The initial step entailed choosing the center sagittal picture (Fig. 1a), wherein (1) the tibial attachment of the PCL, (2) the intercondylar eminence, and (3) the anterior and posterior tibial cortices manifested a concave configuration. Step two entailed placing one cranial and one caudal circle in the tibial head. The cranial circle was required to contact the anterior, posterior, and cranial tibial cortices, whereas the caudal circle needed to engage the anterior and posterior cortical borders. In instances with indistinct boundaries between the cortex and the medullary canal, the midpoint of the transition zone between a distinct black cortex and a pale gray medullary canal was used. The center of the caudal circle was located on the perimeter of the cranial circle to establish a uniform relative distance between the two circles. The MRI longitudinal axis was established by a line linking the centers of the two circles, which was overlaid and remained stationary during the whole picture series (Fig. 1a). Step three entailed locating the MRI depicting the mediolateral centroid of the medial plateau. A tangent to the medial plateau, linking the superior-anterior and posterior cortical boundaries, was illustrated in this illustration. The slope of the medial plateau was determined by the perpendicular line to the MRI-longitudinal axis and the tangent to the medial plateau (Fig. 1b). The lateral plateau PTS was measured at the mediolateral center of the lateral plateau by a tangent to the highest flat region between the superior-anterior and posterior cortices (Fig. 1c) [18].

**Fig. 2:**
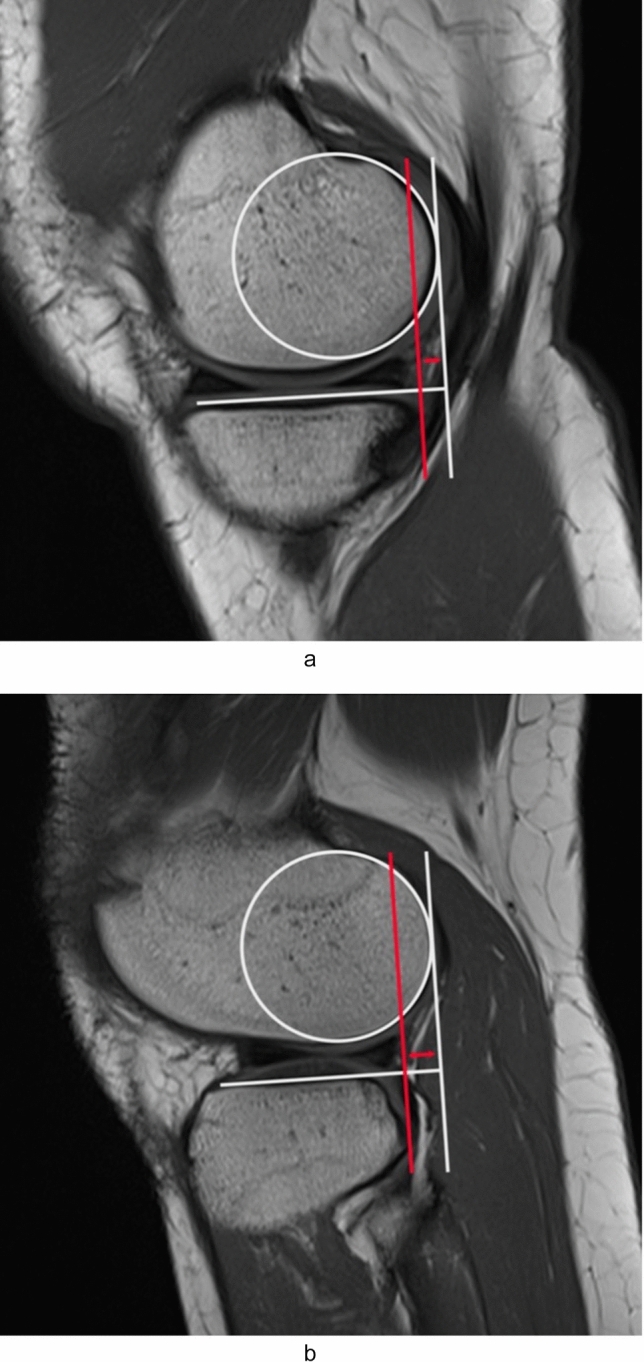
Assessment of anterior tibial translation (ATT) on sagittal T1-weighted knee MRI. A circle is drawn to fit the posterior femoral condyle. Two lines, perpendicular to the tibial plateau, are drawn: one tangent to the circle (white vertical line) and one tangent to the posterior tibial aspect (red line). The distance between these two lines represents the ATT. **a** Medial ATT, assessed at the level of the first image showing the origin of the medial gastrocnemius tendon. **b** Lateral ATT, calculated on the slice at the most medial aspect of the fibula at the tibiofibular joint

For anterior tibial translation, standardized reference points were utilized on sagittal MRI scans to assure measurement consistency. Medial ATT was assessed on the initial MRI picture depicting the origin of the medial gastrocnemius tendon on the femur; lateral ATT was evaluated on the image revealing the most medial portion of the fibula at the tibiofibular joint. A circle was constructed to optimally match the posterior femoral condyle using the sagittal scans. A line orthogonal to the tibial plateau was delineated along the posterior edge of the circle. A supplementary line, orthogonal to the tibial plateau, was delineated in the posterior region of the tibia. The interval between these lines signified the ATT (Fig. 2) [19].

### Statistical analysis

We determined that at least 189 subjects undergoing primary ACL reconstruction were needed to estimate a 40% ± 7% proportion of subjects with a PCL angle of < 105°, a positive indicator of ACL injury, with a confidence level of 95% [17]. Sample characteristics were presented with absolute frequencies and percentages or mean ± standard deviation. Continuous variables were tested for normality using the Shapiro–Wilk test. Radiological scores (PCL angle, medial tibial slope angle, lateral tibial slope angle, medial anterior tibial translation, and lateral anterior tibial translation) were presented overall and categorized by sex, age, and time interval between injury and MRI. Age groups were predefined by dichotomizing age at its mean value, while a cut-off of 90 days was selected to differentiate between chronic and acute lesions. Differences between groups were assessed using t-tests or Wilcoxon-Mann Whitney tests, depending on score distribution. Spearman rank correlations were also estimated to explore correlations among the collected variables.

A two-tailed *p*-value of < 0.05 was considered to indicate statistical significance. All statistical tests were performed using R version 4.3.0 (R Foundation for Statistical Computing, Vienna, Austria. URL https://www.R-project.org/).

## Results

Cohen's kappa showed excellent agreement between the two readers (0.954, *p* < 0.001). The ICC for the reliability of MRI measurements was 0.96 (95% CI 0.94–0.97).

### Demographic data

A total of 193 patients were included in the study, comprising 91 (47.2%) females and 102 (52.8%) males, with a mean age of 30.27 ± 12.54 years. In 109 (56.5%) cases, the left knee was involved, while the right knee was affected in 84 (43.5%) cases. The mean time from injury to MRI was 14.18 ± 55.77 days. Demographic details are shown in Table 1.

**Table 1:** Demographic Data

	N = 193	
	*n (%)*	
Sex		
Female	91 (47.2)	
Male	102 (52.8)	
Side		
Left	109 (56.5)	
Right	84 (43.5)	
	*Mean* ± *SD*	*Median [IQR]*
Age	30.27 ± 12.54	27.00 [21.00, 38.00]
Time interval injury—MRI (days)	14.18 ± 55.77	1.00 [0.00, 2.00]
Age		
< 30 years	110 (57.0)	
≥ 30 years	83 (43.0)	
*Time interval injury—MRI*		
≤ *3months*	*183 (94.8)*	
> *3months*	*10 (5.2)*	

IQR: Interquartile range; MRI: Magnetic Resonance Imaging

### Radiologic results

In the overall population, the mean PCL angle was 128.72 ± 10.33°, the mean medial tibial slope was 3.57 ± 2.33°, and the mean lateral tibial slope was 6.07 ± 3.52°. The mean medial and lateral anterior tibial translations were 4.76 ± 2.02 mm and 7.01 ± 2.48 mm, respectively. Details are shown in Table 2.

**Table 2:** Radiologic Results

	N = 193	*Median [IQR]*
	*Mean* ± *SD*	
PCL angle (degrees)	128.72 ± 10.33	129.00 [122.00, 135.00]
Medial tibial slope angle (degrees)	3.57 ± 2.33	3.00 [2.00, 5.00]
Lateral tibial slope angle (degrees)	6.07 ± 3.52	5.00 [3.00, 9.00]
Medial anterior tibial translation (mm)	4.76 ± 2.02	5.00 [3.00, 6.00]
Lateral anterior tibial translation (mm)	7.01 ± 2.48	7.00 [6.00, 8.00]

SD: Standard Deviation; IQR: Interquartile Range; 4 mm: millimeters

In 190 cases (98.4%), the PCL angle was ≥ 105°, while only three cases (1.6%) were below this threshold.

### Correlations

The PCL angle negatively correlated with medial and lateral anterior tibial translation (*p* < 0.05). Other statistically significant correlations are shown in Table 3.

**Table 3:** Statistical Significant Correlations

Variables		*Correlation*	*p-value*
PCL angle	Medial anterior tibial translation	− 0.26	< 0.001*
PCL angle	Lateral anterior tibial translation	− 0.18	0.012*
Lateral tibial slope angle	Medial anterior tibial translation	0.14	0.047*
Time interval injury—MRI	Lateral tibial slope angle	0.15	0.035*
Age	Medial anterior tibial translation	0.16	0.026*
Medial tibial slope angle	Medial anterior tibial translation	0.21	0.004*
Lateral tibial slope angle	Lateral anterior tibial translation	0.24	0.001*
Medial tibial slope angle	Lateral tibial slope angle	0.32	< 0.001*
Medial anterior tibial translation	Lateral anterior tibial translation	0.37	< 0.001*

^*^Statistical significant value (*p* < 0.05)

PCL: Posterior Cruciate Ligament; MRI: Magnetic Resonance Imaging

### Subgroup analysis

#### Sex

Females exhibited a higher PCL angle compared to males (130.55 ± 10.23° vs. 127.08 ± 10.20°; *p* = 0.019) and lower scores for lateral tibial slope angle, medial anterior tibial translation, and lateral anterior tibial translation (*p* < 0.05). Details are shown in Table 4.

**Table 4:** Subanalysis by gender

	Female	Male	
	N = 91	N = 102	
	*Mean* ± *SD*	*Mean* ± *SD*	*p-value*
Time interval injury—MRI (days)	9.46 ± 44.49	18.39 ± 64.12	0.092
PCL angle (degrees)	130.55 ± 10.23	127.08 ± 10.20	0.019*
Medial tibial slope angle (degrees)	3.82 ± 2.54	3.33 ± 2.11	0.228
Lateral tibial slope angle (degrees)	5.49 ± 3.37	6.58 ± 3.59	0.028*
Medial anterior tibial translation (mm)	4.33 ± 1.73	5.15 ± 2.19	0.009*
Lateral anterior tibial translation (mm)	6.64 ± 2.42	7.35 ± 2.51	0.044*

^*^statistical significant value (*p* < 0.05)

MRI: Magnetic Resonance Imaging; mm: millimeters

### Age

No differences were noted between patients younger and older than 30 years for all radiological scores (*p* > 0.05). Details are shown in Appendix 1.

### Chronic vs. acute

Patients with chronic ACL injuries showed a lower PCL angle compared to those with acute injuries (*p* = 0.032) and a superior grade of lateral anterior tibial translation (*p* = 0.015). Details are shown in Table 5.

**Table 5:** Subanalysis by time from injury to magnetic resonance imaging

	≤ 3 months	> 3 months	*p-value*
	N = 183	N = 10	
	*Mean* ± *SD*	*Mean* ± *SD*	
PCL angle (degrees)	129.09 ± 10.24	121.90 ± 10.16	0.032*
Medial tibial slope angle (degrees)	3.54 ± 2.33	4.00 ± 2.49	0.517
Lateral tibial slope angle (degrees)	5.99 ± 3.53	7.55 ± 3.11	0.134
Medial anterior tibial translation (mm)	4.72 ± 2.04	5.50 ± 1.70	0.147
Lateral anterior tibial translation (mm)	6.90 ± 2.42	9.15 ± 2.89	0.015*

*Statistical significant value (*p* < 0.05)

MRI: Magnetic Resonance Imaging; mm: millimeters

## Discussion

The main findings of this study demonstrate that in the context of ACL injury, the PCL angle has a normal value, particularly in the acute phase, and a direct negative correlation exists between anterior tibial translation (both medial and lateral) and the PCL angle. Furthermore, women demonstrate higher values (130° vs. 127° compared to males).

Our study's result somewhat contrast with the current literature; only three patients reported a PCL angle of less than 105°. Recent literature has indicated that a PCL angle of less than 105° is considered positive evidence for an ACL tear. This discrepancy may be attributed to the fact that nearly all patients evaluated in this study suffered from acute injuries, and the time between trauma and MRI was very short [20].

According to our study, in 1998, Dimond et al. compared MRI findings in 87 patients with acute and chronic ACL tears. The study revealed that increased anterior curvature or bowing of the PCL was more pronounced in cases with chronic ACL tears. The mean value of the PCL bowing ratio for cases with chronic ACL tears was 0.47 compared to 0.37 for cases with acute ACL tears (*p* = 0.013) [21].

Similarly, Sevim et al. examined the impact of prolonged ACL deficiency on the PTS and the sagittal morphology of the proximal tibia, revealing that the knees of patients with chronic ACL deficiency (5 years) exhibited elevated medial and lateral PTS in comparison to the unaffected contralateral knees. Consequently, while strategizing ACL repair for patients with a prolonged history of ACL deficit, it is imperative to precisely assess the preoperative PTS [22].

Numerous research have examined the behavior of the PCL in knees lacking in ACL, utilizing these characteristics as a reference. Gali et al. evaluated the posterior cruciate ligament inclination angle (PCLIA) in MRIs of persons with and without ACL injuries, positing that PCLIA measurement using MRI may function as an adjunctive technique for identifying ACL insufficiency. The scientists concluded that the PCLIA was markedly elevated in people with ACL injuries. The assessment of this angle by MRI imaging may facilitate the identification of ACL insufficiency, hence aiding in a tailored and accurate treatment strategy for ACL injuries [23].

Likewise, Oronowicz et al. sought to ascertain whether the posterior cruciate ligament–posterior cortex angle (PCL–PCA) correlates with the chronicity of ACL rupture, meniscal condition, preoperative knee laxity, or imaging indicators such as the lateral collateral ligament sign or the PTS in knees with ACL injuries [10].

A total of eighty-two participants enrolled in this trial. The median PCL–PCA was 16.2°, with a significant difference between acute (18.4°) and chronic (10.7°) injuries (*p* < 0.01). The median PCL–PCA was markedly reduced by 4.6°. No substantial correlation was identified between PCL–PCA and meniscal condition, PTS, or preoperative anterior knee laxity (Lachman, pivot shift, and ATT measured in millimeters). The findings corroborate the idea that PCL–PCA and the LCL sign may serve as valuable indicators of knee decompensation progression following an ACL injury. [10].

In 2022, Siboni et al. endeavored to verify a novel MRI technique for quantifying the buckling phenomenon of the PCL, indicative of anterior tibial translation, by assessing its reliability and accuracy against established methods in ACL-deficient knees [11]. The evaluation of the curvature of the anterolateral bundle of the posterior cruciate ligament (PCL) was conducted using T2 sagittal MRI slices through three methodologies: (1) the PCL angle (PCLA), (2) the PCL inclination angle (PCLIA), and (3) an innovative approach: the PCL-posterior cortex angle (PCL–PCA), which denotes the angle between the vertical segment of the PCL-ALB and the posterior diaphyseal cortex of the femur. The inter- and intra-observer reliability was assessed for each method. The capacity to differentiate between ACL-deficient and ACL-intact knees was assessed utilizing ROC curves. Compared to previously described approaches, the PCL–PCA was the most dependable and precise technique for assessing the PCL buckling phenomena on MRI in ACL-deficient knees. It provides a straightforward and impartial approach for monitoring ACL-injured patients and is thus advisable for regular use [11].

Analyzing our results, it is possible to highlight a difference in anterior tibial translation between acute and chronic patients. This result aligns with current literature; recently, Cance et al. compared the static ATT value in a control population with that in a population with an isolated ACL injury, reporting a reference static ATT value of 1.31 mm in a non–ACL-injured cohort, which was lower than in the ACL-injured cohort (mean, 2.27 mm) [24].

This anterior lateral tibial translation can have important clinical implications, as reported by Lian et al. The authors determined whether static tibial subluxation, as measured on MRI, is associated with the grade of rotatory knee laxity in ACL-deficient knees [25].

The authors determined that two separate groups exhibiting rotatory knee laxity indicated that static anterior subluxation of the lateral tibial plateau measuring 2.95 mm or more correlated with high-grade rotatory knee laxity, and each millimeter increment in lateral tibial plateau subluxation corresponded to a 1.2-fold increase in the likelihood of high-grade rotatory knee laxity. Anterolateral displacement of the tibial plateau on MRI was not independently correlated with severe rotatory knee laxity when accompanied by a lateral meniscal injury. Preoperative static measures may assist in forecasting high-grade rotatory knee laxity and enhancing the criteria for personalized knee surgery. [25].

Finally, our study showed a statistical difference in PCL angle between males and females; several factors may contribute to this finding. First, as described by Gupta et al. in a cadaveric study, the length of ligaments was found to be greater in males than in females. Thus, in cases of anterior tibial translation, the shorter ligaments in women lead to a different angle compared to males [26].

Another factor relates to differences in laxity associated with gender; Boguszweski et al. confirmed that female knees demonstrated significantly increased laxity and reduced stiffness compared to males [27].

Finally, as reported by Chandrashekar et al., female ligaments exhibit lower mechanical properties when compared to males [28].

### Limitations

This study has limitations. Initially, we did not link the MRI measurement data with clinical or arthroscopic evaluations of the individuals studied, as the diagnosis was solely based on MRI analysis. Nonetheless, the radiologists conducting the assessments evaluated MRI scans of instances with a definitive diagnosis of either damaged or intact ACL. The data included only cases of unequivocally injured ACLs, so removing those that could be classified as partial injuries. Nonetheless, we can investigate the mechanical properties of the residual ACL fibers after partial injuries. Future research is essential to elucidate the characteristics and biomechanical properties of partial ACL injuries, as well as the implications of partial ACL insufficiency.

## Conclusions

In the context of ACL lesions, the PCL angle has a normal value in acute injuries (> 105°) and decreases over time. The PCL angle is negatively correlated with anterior tibial translation, and females have a higher PCL angle compared to males.

## Supplementary Information

Below is the link to the electronic supplementary material.

Supplementary file1 (DOCX 13 kb)

## Data Availability

Raw data are available upon request to the corresponding author.
