# Supplementary material for: The posterior cruciate ligament angle in the setting of anterior cruciate ligament deficient knees: the effect of gender, age, time from injury and tibial slope
Source: Radiol Med. 2025 Jan 25;130(4):534–42. doi: 10.1007/s11547-025-01951-x (PMC12008072; doi:10.1007/s11547-025-01951-x)
Supplement: Supplementary file 1 — Supplementary file1 (DOCX 13 kb) [file 11547_2025_1951_MOESM1_ESM.docx]

**Appendix 1**

**Table S1.** Subgroup analysis for age

|  | **<30 years** | **≥30 years** |  |
| --- | --- | --- | --- |
|  | **N=110** | **N=83** |  |
|  | ***Mean± SD*** | ***Mean± SD*** | ***p-value*** |
| Time interval injury - MRI (days) | 17.86 ±62.02 | 9.30 ±46.12 | 0.317 |
| PCL angle (degrees) | 129.01 ±9.52 | 128.33 ±11.37 | 0.650 |
| Medial tibial slope angle (degrees) | 3.35 ±2.22 | 3.86 ±2.46 | 0.172 |
| Lateral tibial slope angle (degrees) | 5.87 ±3.56 | 6.33 ±3.47 | 0.304 |
| Medial anterior tibial translation (mm) | 4.64 ±2.22 | 4.92 ±1.73 | 0.235 |
| Lateral anterior tibial translation (mm) | 6.76 ±2.32 | 7.34 ±2.67 | 0.057 |
